# Ultrafine Ru nanoparticles integrated on ordered mesoporous carbon for solvent-free hydrogenation of nitroarenes

**DOI:** 10.1039/d3ra03643j

**Published:** 2023-07-12

**Authors:** Hui Liao, Peiqi Weng, Haigen Huang, Ruoxia Tan, Rui Zhu, Yao Liu, Zhijun Wang

**Affiliations:** a Key Laboratory of Coordination Chemistry of Jiangxi Province, School of Chemistry and Chemical Engineering, Jinggangshan University Ji'an Jiangxi 343009 China haigenhuang@126.com wangzhijun@jgsu.edu.cn

## Abstract

The hydrogenation of nitroarenes to aromatic amines with H_2_ under solvent-free conditions in place of organic solvents is a crucial process for the synthesis of fine chemicals. However, the catalysts that have been identified so far are relatively inactive with primary nitroarenes under solvent-free conditions. Herein, ordered mesoporous carbon (OMC)-supported highly dispersed Ru nanoparticles (Ru NPs) were easily prepared by using a coordination-assisted solvent evaporation induced co-assembly method, where 8-hydroxyquinoline exerts a significant influence on the mesostructure ordering and specific surface area of the Ru/OMC composites. The ultrasmall-sized Ru NPs (∼2.0 nm) supported on ordered mesoporous carbon were then applied in the hydrogenation of nitroarenes, exhibiting high activity and selectivity for numerous structurally diverse nitroarenes under solvent-free conditions. Compared with Ru/OMC-imp and Ru/AC-imp, Ru/OMC-800 exhibited a much higher activity and selectivity for hydrogenation of nitroarenes, suggesting its overwhelmingly better performance than OMC or AC supporting noble NPs by simple impregnation method. The strong metal–support interaction is capable of stabilizing the Ru NPs and achieving good recyclability as well as high selectivity.

## Abstract 2

The hydrogenation of nitroarenes to aromatic amines with H_2_ under solvent-free conditions in place of organic solvents is a crucial process for the synthesis of fine chemicals.

## Graphical abstract

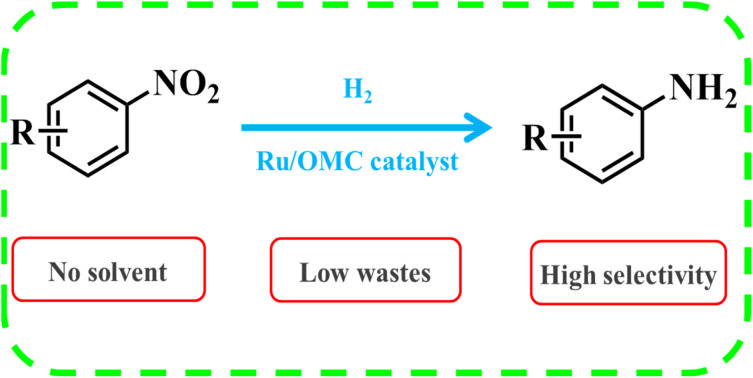

## 1. Introduction

Reduction of nitroarenes is a fundamental laboratory and commercial procedure for the production of aromatic amines, which are important organic intermediates and raw materials widely used in the synthesis of dyes, agrochemicals, pharmaceuticals and various other fine chemicals.^1–3^ The typical reduction routes are carried out using stoichiometric reducing agents such as iron power, zinc powder, tin powder, aluminium powder, sulfur compounds, *etc.*^4–7^ or over transition metal-based catalysts using reducing agents like sodium borohydride, hydrated hydrazine, formic acid, formate, *etc.*, causing serious environmental issues.^8–10^ Catalytic hydrogenation of nitroarenes using molecular hydrogen (H_2_) was regarded as the best choice for the reduction of functionalized nitroarenes because of the clean and efficient production process. To date, much attention has been directed towards the selective hydrogenation of nitroarenes to aromatic amines over supported nano-noble metal catalysts, such as Pt, Pd, Au, and Ru, using H_2_ as a reducing agent.^11–14^ However, these systems often require the use of organic solvents such as methanol, THF, toluene, *etc.* to facilitate heat and mass transfer in liquid-phase reaction systems, but these reagents are expensive and toxic.^15–18^ Nevertheless, the classical hydrogenation catalysts usually generate inevitable byproducts, including nitroso, hydroxylamines, azoxy, and azo derivatives.^19,20^ Besides, the poor chemoselectivity also severely limit the practical applications for hydrogenation of nitroarenes with other functional groups (*e.g.*, –C=C, –COOH, –Cl, –C=O, and –CN).^21^ The most desirable production of aromatic amines is direct and quantitative hydrogenation of nitroarenes over highly efficient solid metal catalysts with hydrogen molecules using green solvents or in the absence of solvent.^22–27^ To date, the development of novel and efficient supported nanometal catalysts remains a big challenge for the green production of aromatic amines.

The metal/OMC composites with a variety of metal nanoparticles that fabricated using traditional impregnation or precipitation techniques have exhibited extraordinary potential applications in hydrogenation catalysis, like Pt/OMC,^28^ Pd/OMC,^29,30^ Rh/OMC,^31^ Au/OMC.^32^ With respect to the catalytic performance, supported ruthenium catalysts are proved to be efficient for selective hydrogenation with higher selectivity especially for halogenated nitroarenes but lower activity than palladium or platinum catalysts.^33–36^ Ordered mesoporous carbon supported ruthenium (Ru/OMC) catalysts are highly favorable due to the combined advantages of the active ruthenium species and a unique OMC support with well controlled porosity and structures, as well as superior acid-base tolerance and hydrothermal stability,^37–39^ which exhibited unusual catalytic properties compared to ruthenium catalysts on the classical supports (Ru/Al_2_O_3_,^40^ Ru/AC,^41–43^ Ru/CNFs^44^ RuP/SiO_2_ (ref. 45) and so on) for hydrogenation reactions. To date, fabricating highly stable Ru/OMC catalysts with ruthenium species homogeneously dispersed on ordered mesoporous carbons with extremely high stability using a facile method remains a challenge, as the Ru/OMC catalysts usually exhibit poor stability due to their property toward aggregation and severe leaching of the ruthenium species, which was caused by their poor dispersion and weak interfacial contact with the OMC.^46^ The reported Ru/OMC catalysts were usually synthesized by traditional impregnation or precipitation on OMC prepared in advance, followed by reduction with hydrogen or other reducing agents. As expected, poor control over the ruthenium nanoparticles dispersion, size distribution and weak attachment onto the surface of OMC were inevitable in this tedious multi-step synthesis method.^47^ From the latest research on the carbon supported metal catalysts, we know that direct pyrolysis of metal organic frameworks (MOFs) or metal coordination polymers under an inert atmosphere can afford an alternative approach that deposit metal nanoparticles firmly attached to a carbonaceous framework.^48–50^ Consequently, it appears particularly significant to directly assemble metal particles onto the OMC frameworks with a plenitudinous interfacial contact and strong interaction, which are formed simultaneously by *in situ* pyrolysis of metal and carbon precursors in one strategy.

Herein, we first develop a facile one-pot coordination compounds assisted co-assembly strategy to synthesize well-defined ordered mesoporous Ru/carbon (Ru/OMC) composites with highly dispersed ultrafine Ru nanoclusters homogeneously-embedded in the carbon matrices *via* the soft-templating method with the assistance of 8-hydroxyquinoline (8-HQ). This facile route renders homogeneously dispersed Ru NPs inside the ordered mesoporous carbons without using any stabilizer and additional reductant and was confirmed by various characterization techniques. Moreover, the obtained Ru/OMC materials showed high efficiency and reusability in solvent-free selective hydrogenation of nitrobenzene to aniline with H_2_ under mild conditions with extremely high stability.

## 2. Experimental

### 2.1. Chemicals

Chemicals and reagents: poly(ethylene oxide)-*block*-poly(propylene oxide)-*block*-poly-(ethylene oxide) triblock copolymer Pluronic F127 (*M*_w_ = 12 600, PEO_106_PPO_70_PEO_106_), RuCl_3_·3H_2_O, phenol, formalin solution (37 wt%), sodium hydroxide, hydrochloric acid, ethanol, and the nitroarenes were purchased from Sinopharm Chemical Reagent Co., Ltd., which were analytical-grade and used as received without purification. All chemicals were used as received without any further purification. Deionizer water was used throughout to prepare all solutions.

### 2.2. General procedure for catalyst preparation

Soluble resol precursors were prepared by using phenol and formaldehyde in a base-catalyzed process according to the procedure reported previously.^51^ The mesoporous Ru/OMC composites were prepared by a one-pot coordination compounds assisted multicomponent co-assembly method using the Pluronic F127, as-prepared 20 wt% resol precursors, ruthenium trichloridetrihydrate (RuCl_3_·3H_2_O) and 8-hydroxyquinoline as the structure directing agent, carbon precursors, metal precursors and coordination agent, respectively. Typically, 1.0 g of Pluronic F127 was dissolved in 14 g of absolute ethanol. Then, 5.0 g of the resol precursor solution (20 wt% in ethanol) was added and stirred for 10 min. Into the above mixture, a certain amount of RuCl_3_·3H_2_O ethanol solution was dropped (3.7 g L^−1^ RuCl_3_·3H_2_O) under constant stirring. Subsequently, required amount of 8-hydroxyquinoline ([8-HQ]/[Ru^3+^] molar proportion in the precursor solution was set as 3.0) was added. After further stirring for 30 min, the mixture solution was transferred onto Petri dishes, self-assembly was occurred followed by evaporation of ethanol for several hours at room temperature in a hood. The resulting sticky films were subjected to thermal polymerization at 100 °C for 24 h. The obtained composite films were scrapped off, followed by calcination in a tube furnace at 800 °C for 3 h under N_2_ atmosphere at a heating rate of 2 °C min^−1^ to decompose the triblock copolymer templates, carbonize the resol precursors, and *in situ* generate metal nanoparticles. Before heating, the tube furnace was purged with N_2_ gas for at least 1 h to remove air. The resulting solid with different metal contents and different calcination temperature were denoted *x*Ru/OMC-*T* composites (*x* is the weight percent of Ru in the catalyst where the Ru content in each sample on the basis of the RuCl_3_·3H_2_O added and the total carbon mass used, wt%; *T* represents calcination temperature, °C). In comparison, the samples 3Ru/OMC-800-imp, 3Ru/AC-800-imp, 3Ru/TiO_2_-800-imp, 3Ru/Al_2_O_3_-800-imp were also prepared by impregnation method.

### 2.3. Catalyst characterization

X-ray diffraction (XRD) patterns were obtained using a Rigaku D/MAX-2200 apparatus with a Cu Kα radiation (*λ* = 0.15418 nm) at a voltage of 40 kV and a current of 40 mA. N_2_ adsorption–desorption carried out using a Micromeritics ASAP 2020 Sorptometer at −196 °C. Before the measurement, the sample was degassed at 200 °C for 10 h. The Brunauer–Emmett–Teller (BET) method was utilized to calculate the specific surface areas (SBET) using adsorption data in a relative pressure range from 0.05 to 0.25. By using the Barrett–Joyner–Halenda (BJH) model, the pore volumes and size distributions were derived from the adsorption branches of isotherms, and the pore size (*D*_p_) was obtained from the maximum of the pore distribution curve. The pore volume (*V*_p_) was taken at *P*/*P*_0_ = 0.990 single point Transmission electron microscopy (TEM) micrographs were performed with a JEOL JEM-2010F field emission microscope operating at 200 kV. The weight percentage of Ru deposited was analyzed by inductively coupled plasma atomic emission spectrometry (ICP-AES).

### 2.4. Catalytic reactions and product analyses

Under industrially viable conditions, we have proposed a new protocol that is also applicable to solvent-free hydrogenation of numerous solid structurally diverse nitroarenes. Though a majority of the nitro compounds are solid under normal temperature, they become liquid after heated to their melting points. During the reaction process, the reactants can act as role of the solvents. Because of that, it is practicable to develop a greener and rapid process for the hydrogenation of these solid nitroarenes in liquid phase under solvent-free conditions. Typically, Selective hydrogenation of nitrobenzene was carried out in a 50 mL high pressure reactor with magnetic stirring. In a typical reaction, the catalysts and substrates were charged into the reactor. Prior to testing, the reactor was flushed five times with 1.0 MPa H_2_ and depressurized to atmospheric pressure. Then, the reaction mixture was heated to selected temperature, and reacted at 4 MPa H_2_ pressure and at a stirring rate of 900 rpm. After the reaction, the remaining hydrogen was discharged slowly at room temperature and ethyl acetate (10 mL) and *n*-decane (100 μL) as standard were added into the reactor. The reaction mixture was separated by filtration and further diluted with ethyl acetate (*ca.* 20 mL) for analysis. The products were identified by gas chromatography-mass spectrometry (GC-MS) and were analyzed on a GC with a capillary column and a flame ionization detector.

## 3. Results and discussion

### 3.1. Structure identification of the *x*Ru/OMC-800 catalysts

**Fig. 1:**
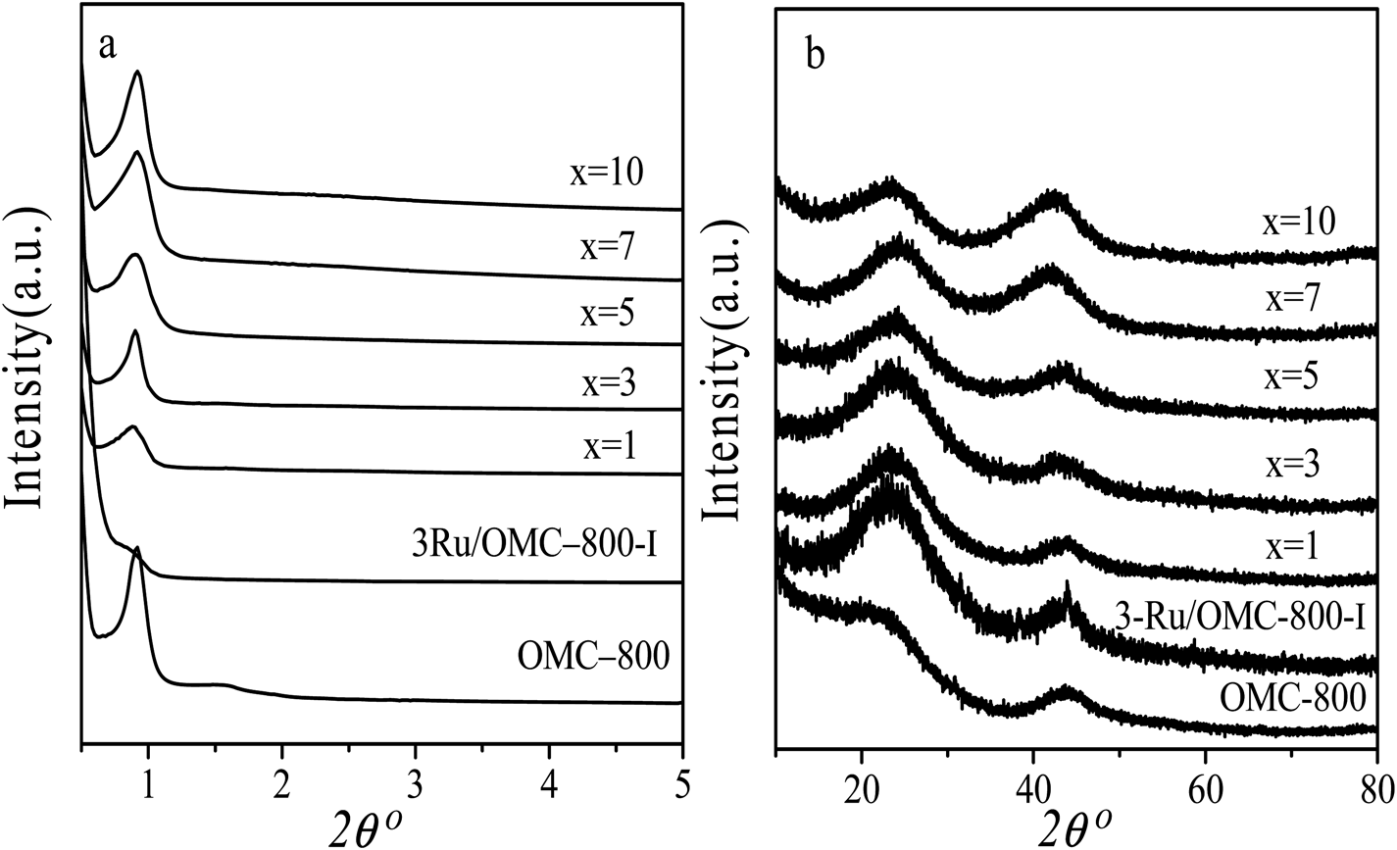
(a) Low-angle XRD and (b) wide-angle XRD pattern of OMC-800, *x*Ru/OMC-800 and Ru/OMC-800-I.

**Scheme 1:**
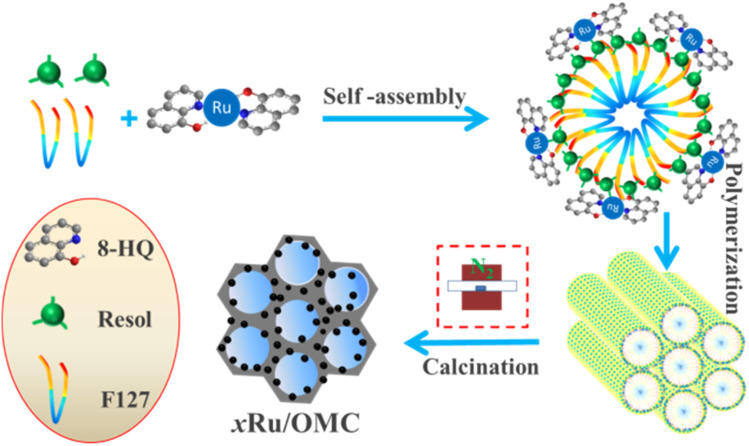
Synthetic strategy for the Ru/OMC catalysts.

The emphasis on the atomic efficiency and green nature of catalysis has set new challenges related to the design and development of highly active, selective, and durable heterogeneous catalytic materials. To fabricate Ru/OMC catalysts with high activity for hydrogenation of nitroarenes, it is critical to the development of novel nanostructures with tunable structure and with ultrafine Ru nanoparticles embedded in the carbon frame with high dispersion to provide more active sites. The Ru content is a critical factor to consider. The structures of the prepared carbon and Ru/carbon samples were first analyzed by XRD. As the low angle XRD patterns of *x*Ru/OMC-800 (*x* = 0, 1, 3, 5, 7, 10) components and the sample prepared without adding 8-HQ (3Ru/OMC-800-I) presented in Fig. 1a. Most of the samples show three peaks in the low angle region, a sharp diffraction peak at around 0.9°, and two weak peaks between 1.7° and 2.1°, which can be indexed as reflections of (100), (110), and (200) planes corresponding to a two-dimensional (2D) hexagonal (*P*6*mm*) structure. While in the case of the sample without 8-HQ in the reaction solution, the corresponding diffraction peak was rather weak, which is indicative of the weak ordering of the mesoporous structure. On the other hand, highly ordered mesoporous carbon with a 2D hexagonal structure (*p*6*mm*) under similar conditions in the absence of ruthenium chloride could be obtained. This result demonstrated that the addition of ruthenium chloride inhibit the self-assembly process of resol and F127 in the solvent evaporation process, leading to carbonaceous frameworks with disordered and lack uniform mesoporous arrays. The formation of the controlled ordered mesoporous carbon with a two-dimensional (2D) hexagonal (*P*6*mm*) structure is mainly attributed to the strong chelating effect of 8-HQ molecules, as the process for the preparation of the catalysts *x*Ru/OMC-*T* schematically illustrated in Scheme 1. In our study, 8-HQ was first used as role through chelation with metal ions and interaction with the phenolic hydroxyl groups of resol molecules that serve as a bridge interact with the EO segments of Pluronic F127 template *via* hydrogen bonding with surfactant F127 *via* electronic attraction in the ethanol solution phase, facilitating the formation of a F127 directed two-dimensional (2D) hexagonal (*P*6*mm*) ruthenium–8-HQ–resol–F127 mesophase. During the self-assembly process, high dosage of 8-HQ not only binds and disperses Ru^3+^ well in sol–gel forming process but also promoting the formation process of a dark-blue hexagonally arranged F127 micelles associated with resol and Ru^3+^ ions spontaneously assemble into a highly ordered mesostructured upon continuous evaporation of ethanol. Owing to the difference in chemical and thermal stability between resol and surfactant F127, as well as the reducibility of carbons produced *in situ* by carbonization of resol, the following pyrolysis is a multi-utilization strategy to remove F127, carbonize resol, and reduce metal ions in a single step. During pyrolysis, F127 framework acts as a soft template to consolidate the two-dimensional (2D) hexagonal (*P*6*mm*) structure.

Textural parameters and mesoscale ordering of the materials were further analyzed using nitrogen adsorption–desorption. Nitrogen sorption isotherms and pore size distribution curves are shown in Fig. 3. It can be seen that all the samples show typical type IV isotherms with a sharp capillary condensation step at *P*/*P*_0_ = 0.4–0.8 and a well-defined H_1_-type hysteresis loop, indicating the mesoporous structures of materials with cylindrical channels. The pore size distributions are very narrow with peaks arranging from 3.5 nm to 4 nm. Some typical textural parameters of the samples are listed in Table 1. The BET specific surface area of the samples is calculated to be 530–640 m^2^ g^−1^ with a narrow fluctuation for the *x*Ru/OMC-800 (*x* = 0, 1, 3, 5, 7, 10). The decrease of the surface area without adding 8-HQ indicates the structural shrinkage caused by high-temperature pyrolysis.

**Fig. 2:**
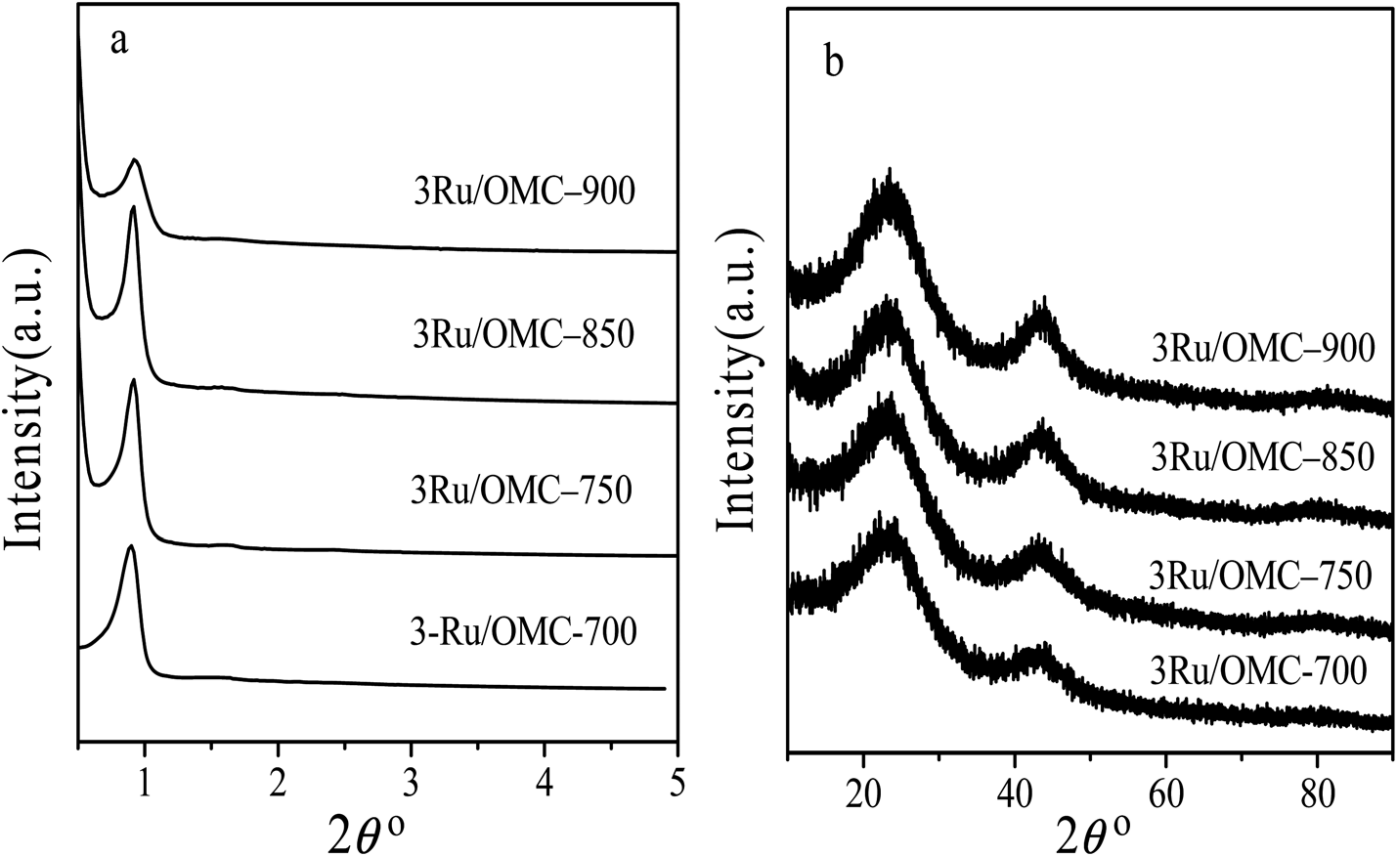
(a) Low-angle XRD and (b) wide-angle XRD pattern of 3Ru/OMC-700, 3Ru/OMC-750, 3Ru/OMC-850 and 3Ru/OMC-900.

**Fig. 3:**
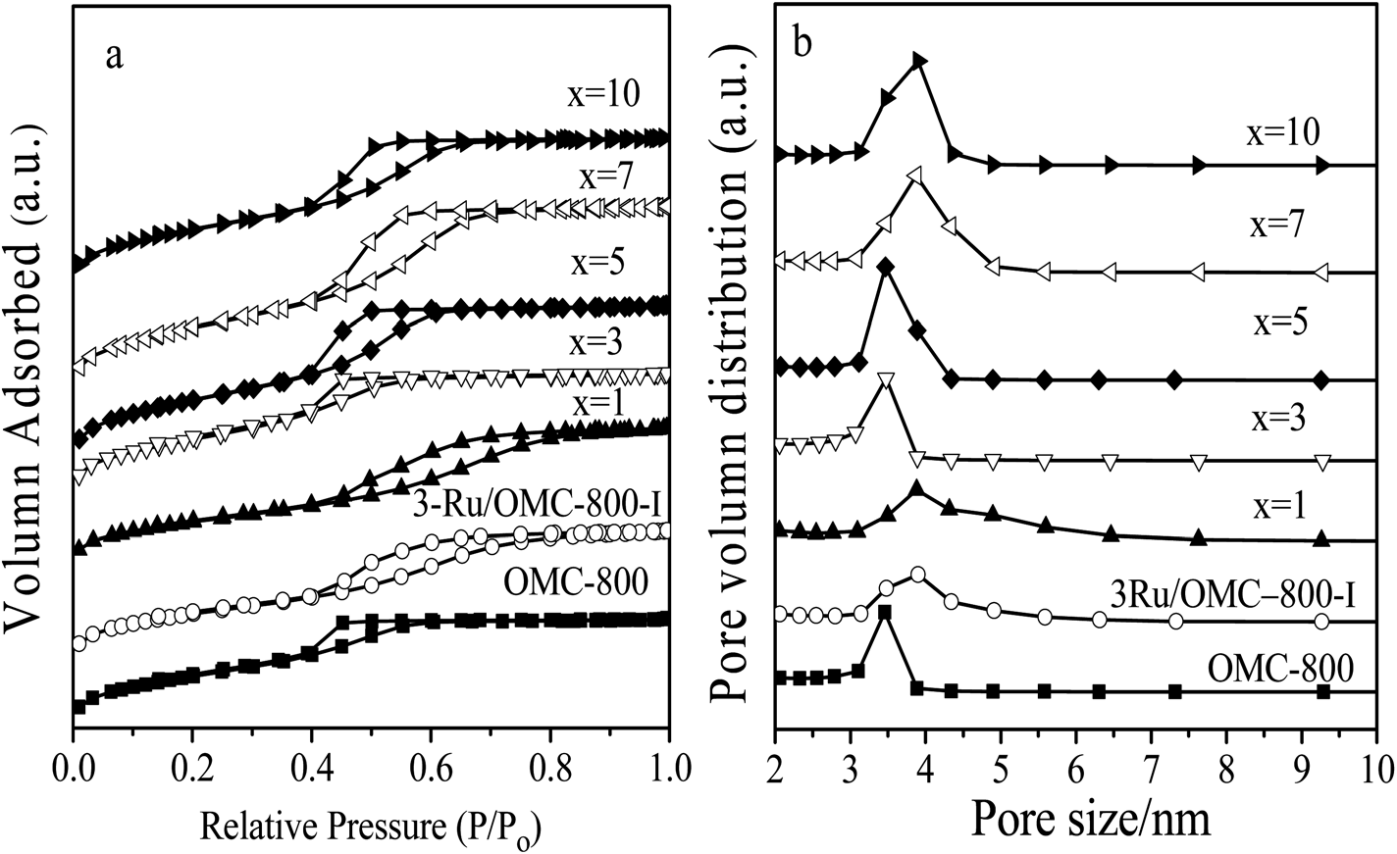
(a) N_2_ sorption isotherms, and (b) BJH pore size distributions of the OMC-800, *x*Ru/OMC-800 and Ru/OMC-800-I.

**Table 1:** Ru contents, structural and textual properties of the *x*Ru/OMC-*T* composites

Sample	Ru^a^ (wt%)	*a*_0_ b (nm)	*S*_BET_ (m^2^ g^−1^)	*V*_p_ (cm^3^ g^−1^)	*D*_p_ (nm)	*W*_t_ c (nm)
OMC-800-3	—	12.3	531.1	0.36	3.5	8.8
3Ru/OMC-800-I	2.11	—	559	0.41	3.8	—
1Ru/OMC-800	1.06	15.8	572.3	0.49	3.8	12
3Ru/OMC-800	2.68	12.6	613	0.40	3.5	7.5
5Ru/OMC-800	4.41	12.9	639.3	0.47	3.5	9.4
7Ru/OMC-800	6.43	12.3	627.7	0.51	3.8	8.5
10Ru/OMC-800	9.72	12.3	586.1	0.44	3.8	8.5
3Ru/OMC-700	2.39	11.3	587.2	0.37	3.5	7.8
3Ru/OMC-750	2.52	12.3	642.1	0.43	3.5	8.8
3Ru/OMC-850	2.44	12.3	635.2	0.44	3.5	8.8
3Ru/OMC-900	2.40	12.3	495.2	0.31	3.2	9.1

a Ru contents were determined by ICP.

b Calculated from SXRD patterns (*a*_0_ = 2*d*_100_/√3).

c Wall thickness values calculated from *W*_t_ = a_0_ – *D*_p_.

TEM images of the Ru/OMC (Fig. 5a–h) show stripe-like and hexagonally arranged patterns in large domains, further confirming an ordered 2-D hexagonal mesostructured, except for the sample prepared without adding 8-HQ, which show disordered structure. As shown in TEM images, highly ordered hexagonal arrangements of pores along the [001] direction is observed clearly. The uniform, highly ordered stripe-like and hexagonally arranged images in large domains indicate a highly ordered mesostructured, which was consistent with the result of low-angle XRD patterns and made the ordered mesoporous carbon structure of samples conformed. In addition, the superfine uniform metal nanoparticles and small pore diameter were apparent in the TEM images. Interestingly, the average particle size of the Ru NPs kept a slight change at approximately 1.8–2.5 nm when the ruthenium concentration varies; while the dispersion of uniformly distributed Ru NPs improves as the molar percentage of Ru decreases. This could be attributed to the carbon *in situ* generated by carbonization of resol acts as a building block to confine and enclose the growth of Ru nanoparticles during carbonization process. Owing to the strong coordination or dispersion effect of 8-HQ species during the coassembly process and effectively restrain the crystal growth through effectively coordinating Ru preventing the aggregation during pyrolysis even under high temperature. So in this way, well-dispersed and homogeneous metallic Ru nanoparticles were incorporated into mesoporous carbon matrices through a simple one-pot synthesis procedure with very accessible hydrothermal conditions.

As wide-angle XRD (Fig. 1b) was not informative to determine the electronic state of the Ru element due to the very small size of the NPs, which only exhibited two remarkable broad diffraction peaks at 2*θ* values of approximately 23.5° and 43.5°, corresponding to the (002) and (101) diffractions of typical amorphous carbon, respectively. Further insight into the catalyst was obtained by XPS (Fig. 7): The corresponding spectra of carbon, ruthenium and oxygen for the surface of the 3Ru/OMC-800 composite as a case were directly and distinctly detected by the XPS elemental survey scans. For the sake of determining the electronic state of Ru in the 3Ru/OMC-800 catalyst, what we used is the high-resolution of the XPS measurement, which is high enough to make a distinction between them and confirm the electronic state of Ru in spite of the sectional germination between the peaks of C1s and Ru 3d. As shown in Fig. 7, the peaks at 284.6, 285.2 and 281.1 eV suggest the presence of C1s, Ru3d_3/2_ and Ru3d_5/2_, respectively. The binding energy of the Ru3d_5/2_ level in the 3Ru/OMC-800 catalyst is about 0.9 eV higher than that of the standard zero-valent state of Ru (280.2 eV), suggesting that the Ru^3+^ ion has been successfully reduced.^52^ The shift indicates that the electron transfer may occur between the oxygen and the Ru nanoparticles of the 3Ru/OMC-800 composites, and as expected, the electron density of the Ru atoms is attracted and reduced by the high electronegativity of oxygen, which lead to an electron-deficient state of electron deficiency of the Ru nanoparticles for the 3Ru/OMC-800 composites.^53^ In addition, the other C1s spectra of 3Ru/OMC-800 composites could be estimated to be C–OH, C (epoxy/alkoxy), and C=O groups and their peaks were at 285.8, 287.7 and 289.6 eV, respectively.^54^

**Fig. 4:**
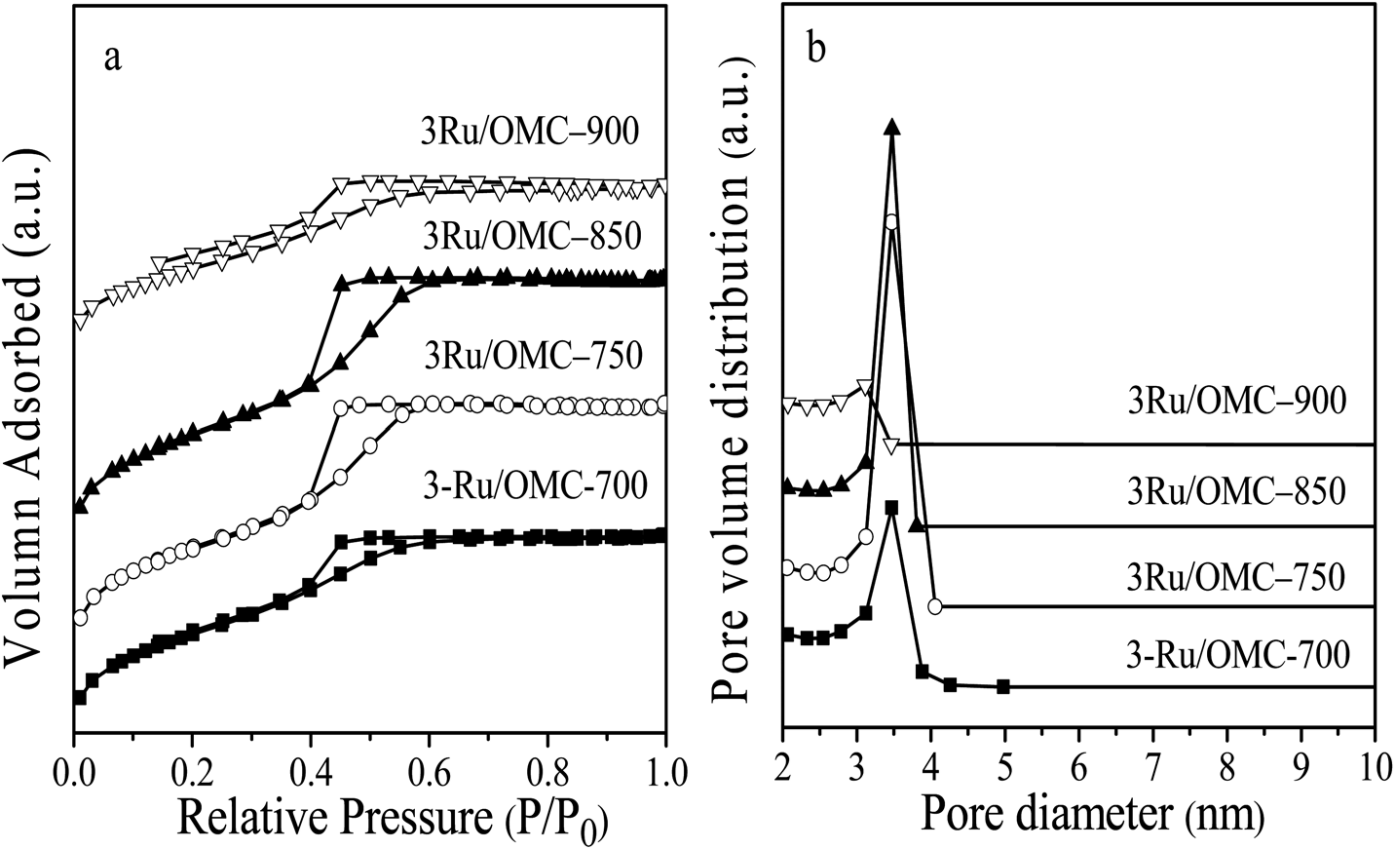
(a) N_2_ sorption isotherms, and (b) BJH pore size distributions of the 3Ru/OMC-700, 3Ru/OMC-750, 3Ru/OMC-850 and 3Ru/OMC-900.

The key idea of our method for synthesis of the *x*Ru/OMC-800 was to exploit the efficient growth of ultrafine Ru nanoparticles embedded in the carbon frame with high dispersion to provide more active sites. Obviously, smaller Ru nanoparticles with higher active surface should be beneficial for the deposition of ultrathin Ru nanoparticles and thereby for enhanced catalytic activity. Thus, we subsequently studied the effect of pyrolysis temperature on the properties of resultant products, as pyrolysis temperature plays a decisive role in particle size. The dispersion of the Ru/OMC catalyst was determined with XRD and electron microscopy techniques. The wide-angle patterns of 3Ru/OMC-*T* (*T* = 700, 750, 850, 900) in Fig. 2b also reveal that there are two broad peaks lied in around 23.5° and 43.5°, indication of (002) and (101) diffractions of typical amorphous carbon correspondingly, and because there were also no obvious peaks of Ru, it is deduced that the Ru nanoparticles in the samples of 3Ru/OMC-*T* were very small. As expected, the surface areas and pore volumes of the 3Ru/OMC-T composites decreased with increasing calcination temperature probably due to the blocking of the mesostructure assembling, although a partial pore blocking cannot be ruled out (Table 1). The morphology of all 3Ru/OMC-*T* (*T* = 700, 750, 850, 900) samples was observed by TEM images (Fig. 6a–d) and the last three pictures demonstrated stripe-geometry and hexagonally arranged pore morphology, which was consistent with the result of low-angle XRD patterns (Fig. 2a) and made the ordered mesoporous carbon structure of samples conformed. In addition, the superfine uniform metal nanoparticles (∼2 nm) and small pore diameter were apparent in the TEM images. A more detailed pore structural characterization was revealed by N_2_ sorption–desorption isotherms in Fig. 4, and all 3Ru/OMC-*T* samples show typical type-IV carves with a typical hysteresis loop, revealing the appearance of apparent mesoporous structure in synthesis. Structural and textual properties of 3Ru/OMC-*T* (*T* = 700, 750, 850, 900) composites were listed in Table 1, which showed the similar surface areas and pore parameters. The decrease of the pore diameters and the unclosed hysteresis loop of 3Ru/OMC-900 resulted from the structural shrinkage caused by high-temperature pyrolysis. These N_2_ adsorption/desorption data are in good agreement with the small angle XRD and TEM results.

**Fig. 5:**
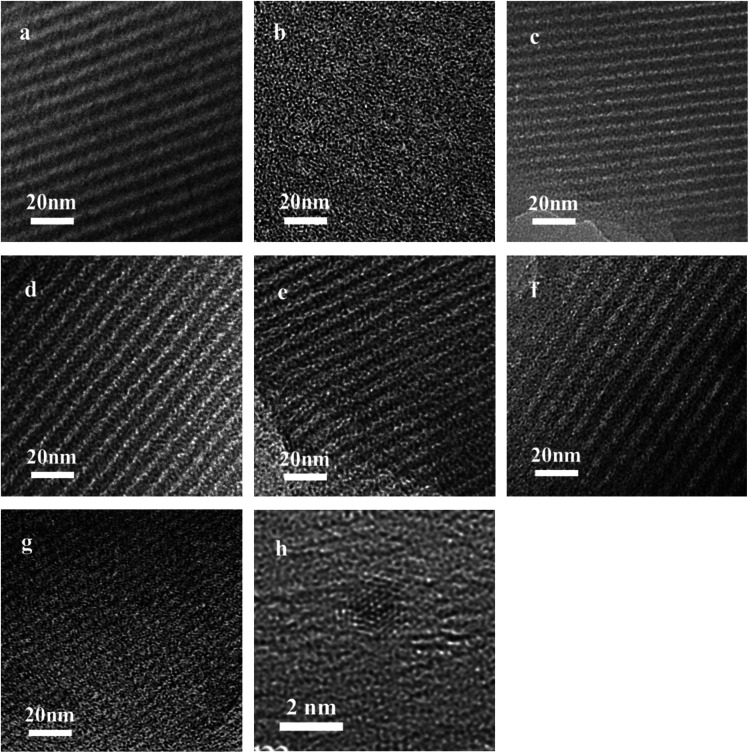
TEM images of the OMC-800, *x*Ru/OMC-800 and Ru/OMC-800-I: (a) OMC-800, (b) Ru/OMC-800-I, (c) *x* = 1, (d) *x* = 3, (e) *x* = 5, (f) *n* = 7, (g) *n* = 10 and (h) HRTEM images of the 3Ru/OMC-800.

### 3.2. Catalytic reaction

For nitrobenzene (NB) selective hydrogenation, ruthenium catalysts were proved to be good catalysts for restraining side reaction, while maintaining high efficiency for reduction of the nitro group. The catalytic properties of the Ru/OMC catalyst were probed using the selective hydrogenation of nitrobenzene (NB) as a model reaction. Table 2 summarizes the conversions of NB over the *x*Ru/OMC-*T* composites for the NB hydrogenation for 2 h for comparison. The pristine OMC was almost inactive for this reaction, same with the result without catalyst (Table 2, entry 1, 2). It could be seen that the NB conversions increased with the Ru content in the absence of solvent (Table 2, entries 4–8) at first, while with the further increasing of the Ru content, compared to the 3Ru/OMC-800, the TOF of higher Ru contents samples decrease from 633 to about 437, even higher conversions obtained after reaction with the same time. Further proof can be obtained by controlled trials (not shown here) with the same amount of Ru addition of the samples based on the actual Ru contents analyzed by ICP. The catalytic efficiency declined by further increasing the Ru contents, mainly due to the larger particle size and the decreasing in Ru dispersion degree. It should be noted that the addition of appropriate amount of 8-HQ, can dramatically improve the conversion from 41.1% (Table 2, entry 3) to 86.6% (Table 2, entry 5), indicating that the activity of the catalyst prepared with the assistance of 8-HQ obtained a multiplying increase. Because of restricted use of 8-HQ, the perturbation from the Ru(iii) ions during process of synthesis could affect structure and the catalytic activities of catalysts. In accordance with the TEM results that weak ordering of the mesoporous structure and larger Ru particles was obtained for the sample 3-Ru/OMC-800-I than the 3Ru/OMC-800. The particle size has a significant effect on hydrogenation reactions. The average diameter of the Ru nanoparticles in the Ru/OMC catalyst is smaller than that of the Ru/CNTs (4 nm) and Ru/CNFs (3 nm) catalysts with similar Ru contents reported by Oubenali *et al.*^55^ Previous research has shown that formation of weakly bonded subsurface hydrogen is the key factor for the hydrogenation reaction to occur. The accessibility of these hydrogen atoms is enhanced on the highly dispersed metal nanoparticles as a result of their nanoscale dimensions.^56,57^ Pyrolysis temperatures also play a key role in the performance of catalyst. As illustrated in Table 2, entry 5, 9–12, compared to the 3Ru/OMC-700 and 3Ru/OMC-750 samples, 3Ru/OMC-800 prepared by pyrolysis temperature higher exhibit higher activity, the conversion increased from 51.3%, 66.8% to 88.6%, which may be caused by the carbon matrix with more highly active functional group. Further increasing the pyrolysis temperature, a decrease in activity was observed. This could inculpate to the decrease of the specific surface area and pore diameters with increasing the pyrolysis temperature indicates the structural shrinkage caused by high-temperature pyrolysis, leads to a significant decrease in the activity of the catalyst. As the reaction rate is also determined by the mass transfer limitations between the reactants and solid catalyst. Through prolonging the reaction time up to 2.5 h, 100% conversion and 100% selectivity can be achieved for all the samples, suggesting great potential in chemoselective reduction of functionalized nitroarenes.

**Table 2:** Catalytic performance of various supported catalysts for chemoselective hydrogenation of nitrobenzene^a^

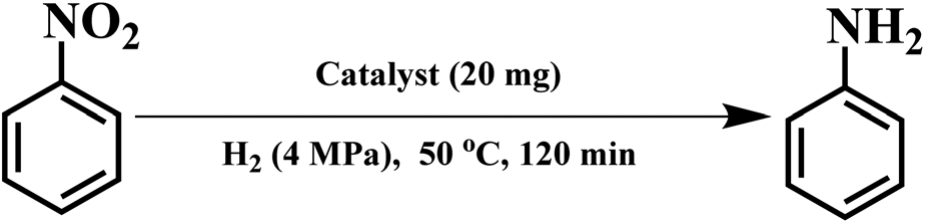				
Entry	Catalyst	Conv. (%)	Sel. (%)	TOF
1	No catalyst	0	0	0
2	OMC	0	0	0
3	3Ru/OMC–800-I	41.1	92.0	206
4	1Ru/OMC–800	15.4	91.7	77
5	3Ru/OMC–800	86.6	95.7	633
6	5Ru/OMC–800	99.7	99.0	498
7	7Ru/OMC–800	99.6	98.6	454
8	10Ru/OMC–800	99.3	99.1	437
9	3Ru/OMC–700	51.3	94.7	406
10	3Ru/OMC–750	66.8	89.3	488
11	3Ru/OMC–850	70.4	90.5	517
12	3Ru/OMC–900	67.8	92.4	489
13	3Ru/OMC–800-imp	44.1	73.2	314
14	3Ru/OMC–800-imp	62.9	57.4	476
15	3Ru/AC–800-imp	23.6	52.8	202
16	3Ru/TiO_2_-imp	19.7	63.1	198
17	3Ru/Al2O_3_-imp	34.2	48.3	248

a Reaction conditions: 20 mg catalyst, NB/Ru = 1000/1 (mol mol^−1^), H_2_ pressure = 4.0 MPa, 50 °C, 120 min.

To understand the benefits of highly dispersed Ru/OMC catalyst with Ru nanoparticles strongly attached to the well-defined carbonaceous framework for the hydrogenation of NB, the 3Ru/OMC-imp catalysts with 3.0 wt% loading were also prepared by simply impregnation method and used for the hydrogenation of NB under the same conditions, which was also baked under the temperature of 800 °C. Compared to the sample synthesized by one-pot method, the Ru/OMC-imp catalyst showed much lower activity, only 44.1% (Table 2, entry 13) conversion was obtained under same reaction conditions. Significantly, under the same impregnation method, a certain degree of improvement of activity for the catalyst was found by the introduction of 8-HQ (Table 2, entry 14). It is further explained that 8-HQ plays an important role in promoting catalyst activity. As the common method for maximizing the number of active sites and efficiently utilizing expensive metals is to reduce the catalyst particle domain size as far as possible, while further investigation have in fact shown that noble metal deposited on carbons by simply impregnation method can easily leach during catalytic processes because the interaction between the metal NPs and the carriers surface is weak.^58^

Besides, supports can play an important role in the catalytic process by providing new active sites and may strongly affect both the physical and chemical properties of metal nanoparticles. The most-used activated carbon (AC) supporting Ru (3Ru/AC-imp), 3Ru/TiO_2_-imp, and 3Ru/Al_2_O_3_-imp sharing the same mass percentage of 3 wt% Ru were prepared with the same approach, using the hydrogenation of NB as a model reaction. The catalytic activities of the Ru nanoparticles supported on different carriers were tested and compared with the Ru/OMC prepared by the one pot 8-HQ assisted method, On the basis of our experimental data showed in Table 1, the 3Ru/AC, 3Ru/TiO_2_ and 3Ru/Al_2_O_3_ show much lower activity and selectivity to the NB hydrogenation with H_2_ in the absence of solvent (Table 2, entry 15–17). Compared with these supports, the ordered mesoporous carbon with high surface area and large pore volume can favor mass transfer and show better adsorption property. Furthermore, the catalytic activity per surface metal atom and selectivity can depend strongly on the choice of support, and the extent of metal reduction. Besides, the dispersion states and the surrounding environment of central metal or metal oxides can significantly influence the reactivity and selectivity of nanostructured supported catalysts.^59^ On the other hand, the functional group of the carbon matrix also worth considering as previously research have found that the carbonyl group, the carboxylic group, or anhydride has remarkable effects on nitrobenzene hydrogenation.^60,61^ Among these groups which took great parts in the reaction, carbonyl group was highly active, which can be detected, while carboxylic group as well as anhydride can inhibit catalytic activity significantly, which were undetectable form the XPS result in Fig. 7. Modern views suggest that the reaction occurs mainly between reactants chemisorbed on the support and hydrogen activated on the metal sites.

**Fig. 6:**
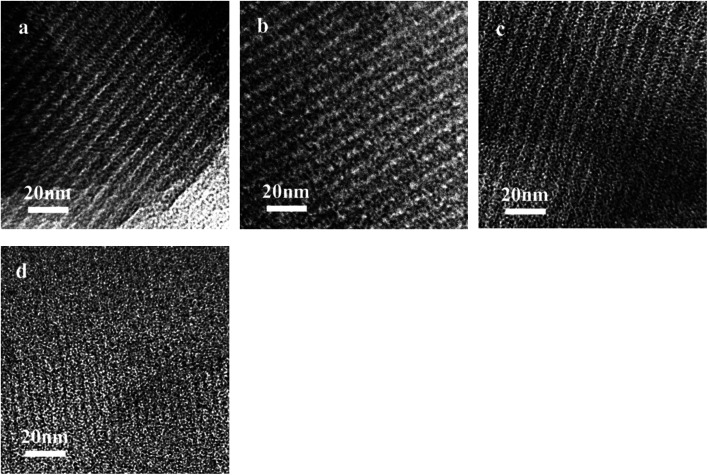
TEM images of the (a) 3Ru/OMC-700, (b) 3Ru/OMC-750, (C) 3Ru/OMC-850 and (d) 3Ru/OMC-900.

**Fig. 7:**
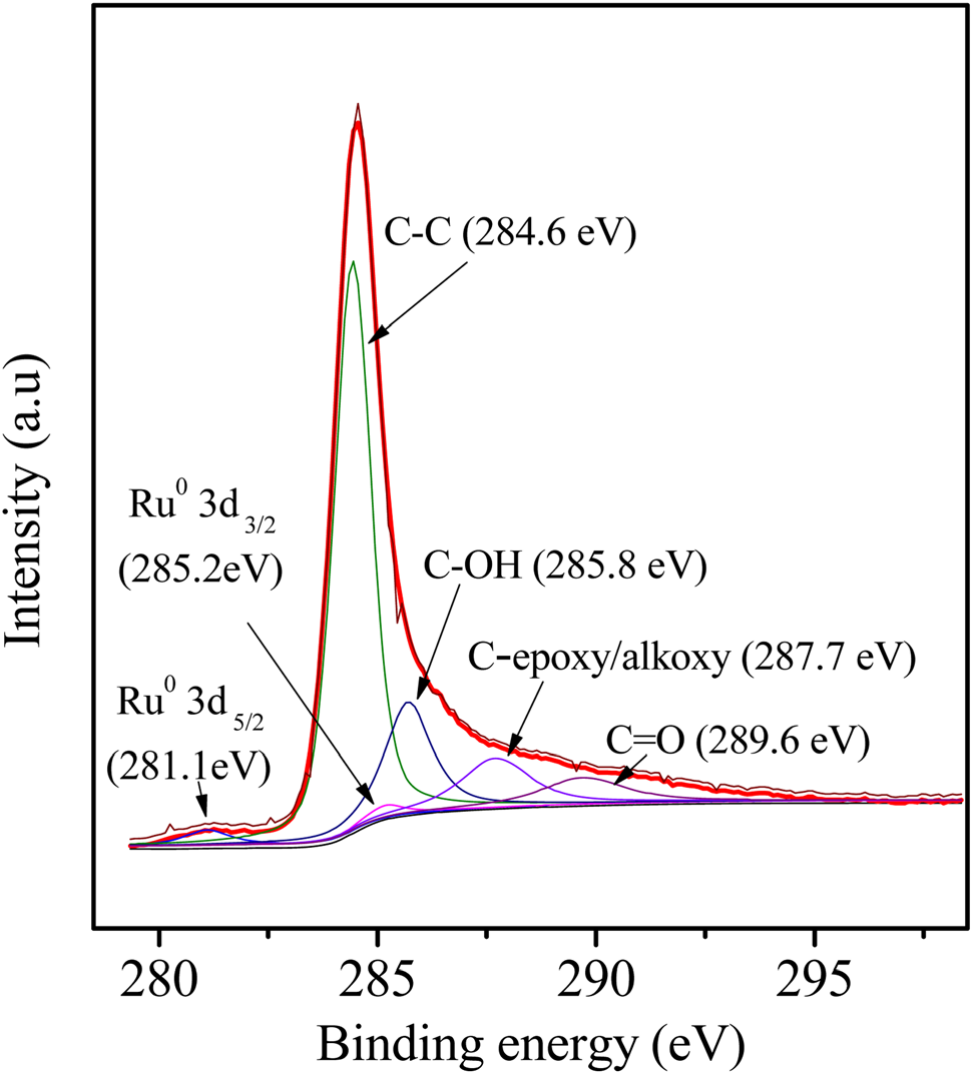
XPS pattern of 3Ru/OMC-800 catalyst.

**Fig. 8:**
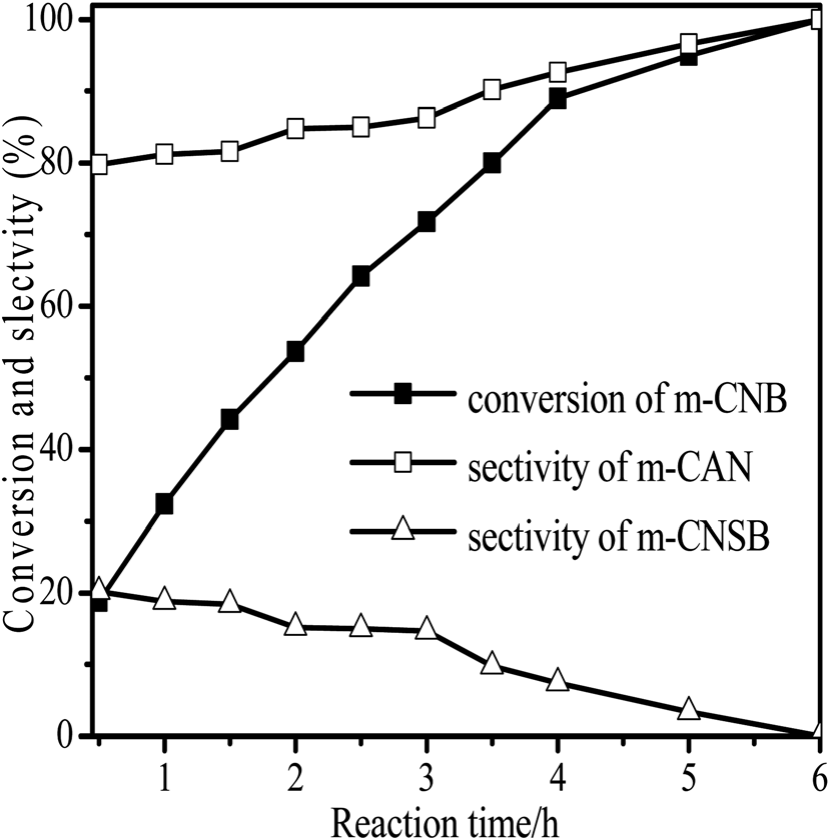
Reaction profiles of 3Ru/OMC-800 as a function of time for the hydrogenation of m-CNB. Reaction conditions: 20 mg catalyst, m-CNB/Ru = 1000/1 (mol mol^−1^), H_2_ pressure = 4.0 MPa, 70 °C.

The performance for reduction of halogenated nitroarenes is an indicator that cannot be ignored to evaluate the catalyst. As for halogenated nitroarenes selective hydrogenation, the hydrogenolysis of the C–X, in which X represents halogen, always happens, especially for Pd, Pt, Ni -based catalysts, while ruthenium catalysts were considered to be potential catalysts for restraining dehalogenation, while maintaining high efficiency for reduction of the nitro group. In order to understand the performance of the catalyst for hydrogenation of halogenated nitro compounds, the catalytic properties of the 3Ru/OMC-800 catalyst were probed using the selective hydrogenation of 3-nitrochlorobenzene (m-CNB) as a model reaction. As illustrated in Fig. 8, the m-CNB conversion increased almost linearly in the former part of the reaction, then gradually slowed down and finally m-CNB was completely converted to the corresponding m-CAN either in the presence or the absence of a solvent. During the entire reactions, there was only a certain amount of m-CNSB accompanied in products, and disappeared with a totally conversion of m-CNB into m-CAN. Interesting, the possible byproducts reported in chlorine substituted nitroarenes reduction, which include the dehalogenated product–aniline, or the coupling product such as *N*-ethylchloroaniline, bischlorophenyldiazene and dichloroazoxybenzene were undetectable in the whole process.^62^ This phenomenon indicated that the nitro group in the aromatic ring was first reduced to the nitroso group, and further was transformed into the corresponding amine in two consecutive steps. During the reaction, undesired hydrodechlorination were avoided, as the –Cl bond remained intact. It is well known that highly dispersed Ru nanoparticles in the channel of OMC would offer more active sites for the hydrogenation, which not only increases the adsorption probability of the reactant molecules for speeding up the reaction but also greatly shortens the residence time of the reaction species and thus avoids over hydrogenation. Moreover, it is generally recognized that the side reaction of hydrodehalogenation of aromatic halides could be inhibited when the metal nanoparticles are situated in electron-deficient states, the extent of electron feedback from the Ru particles to the aromatic ring in m-CAN were weakened.^60^ Take a note of the XPS result the 3Ru/OMC-800 catalyst, when compared to the standard zero-valent state of Ru (the binding energy of the Ru3d_5/2_ level: 280.2 eV), a much higher value (281.1 eV) was obtained in the 3Ru/OMC-800 catalyst, suggesting that the Ru particles in the 3Ru/OMC-800 catalyst presented a much more electron-deficient state. Therefore, it is worth pointing out the high selectivity for hydrogenation of m-CNB over the 3Ru/OMC-800 catalyst mainly attributed to the electronic effect caused by the electron-deficient state of the Ru nanoparticles.

The reaction protocol was further extended to various substituted nitroaromatics to verify the scope, activity, chemoselectivity and the general applicability of hydrogenation reactions catalysed by 3Ru/OMC-800. It could be seen that the 3Ru/OMC-800 showed high activities and excellent selectivity for the hydrogenation of the studied substrates containing electron-donating groups or electron-withdrawing groups like –CH_3_, –CF_3_, –F, –Br, CH_3_O–, –OH, –NH_2_, –NO_2_ and so on, which can be smoothly reduced to the corresponding aromatic amines in the absence of solvent (Table 3, entries 1–19). For example, fluoronitrobenzene or bromonitrobenzene can be smoothly hydrogenated to the desired product quantitatively and the side reaction of dehalogenation reaction was suppressed at complete conversions. Significantly, nitro group in the presence of a broad spectrum of substitutes containing the easily reducible –CHO, –COOH and –CN functional group also remained unaffected during the reaction (Table 3, entries 20–22). For all substrates contain easily reducible groups, the selectivity to functionalized anilines was ≥99% to produce the fully hydrogenated amine. Notably, the catalyst could also promote the heterocyclic nitroaromatic reduction in the yields of >99% without being deactivated by N heterocyclic (Table 3, entries 23–25).

**Table 3:** Selective hydrogenation of various substituted nitroarenes to corresponding anilines over the 3Ru/OMC–800^a^

Entry	Substrate	*T* b (°C)	Time (h)	Conv. (%)	Sel. (%)
1	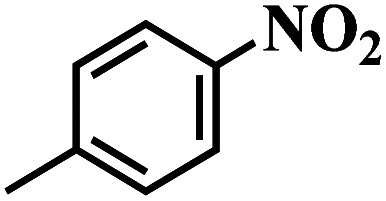	75 (52)	2	>99	>99
2	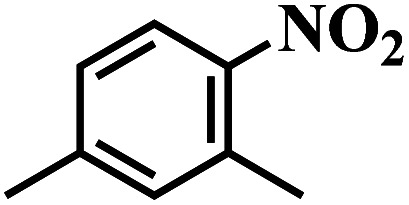	45 (2)	9	>99	>99
3	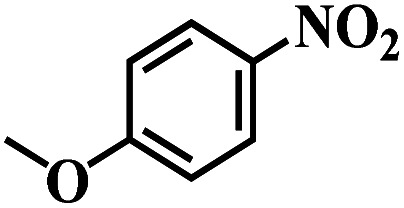	70 (54)	4.5	>99	>99
4	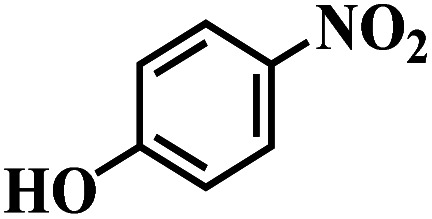	130 (112)	5.5	>99	>99
5	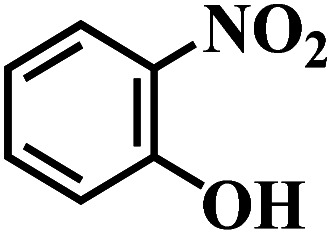	60 (43)	6	>99	>99
6	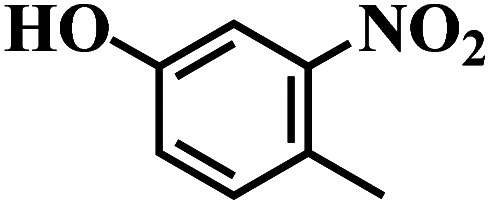	140 (126)	15	>99	>99
7	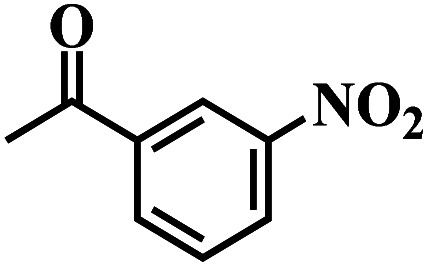	85 (81)	3.5	>99	>99
8	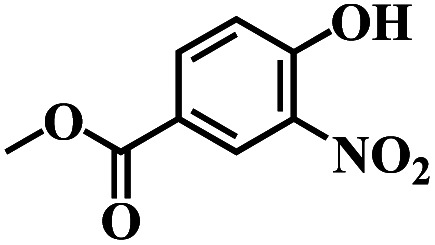	100 (76)	8.5	>99	>99
9	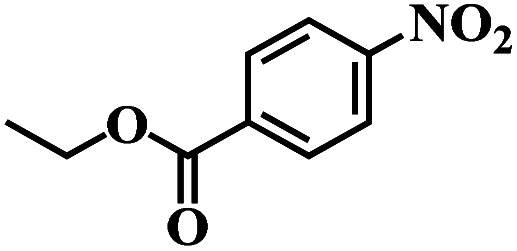	70 (57)	9	>99	>99
10	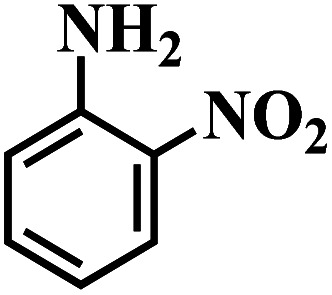	100 (69.7)	3.5	>99	>99
11	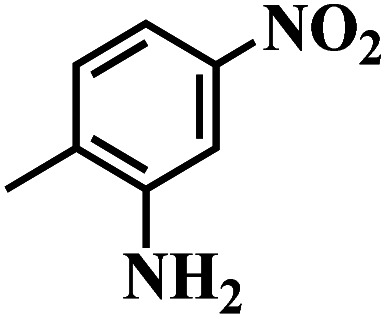	140 (126)	14	>99	>99
12	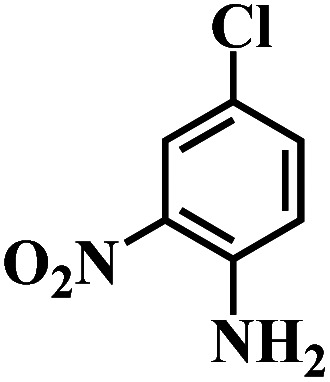	140 (119)	8	>99	>99
13	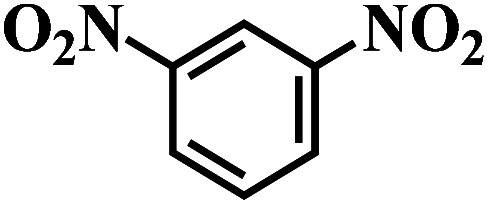	100 (89)	5	>99	>99
14	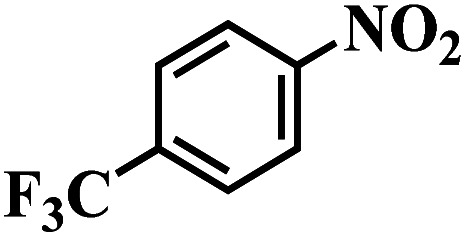	70 (40)	2.5	>99	>99
15	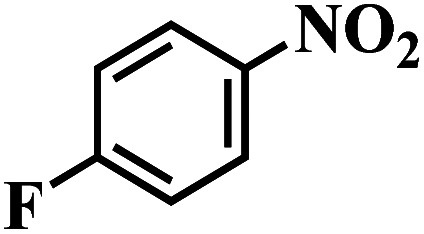	50 (21)	3	>99	>99
16	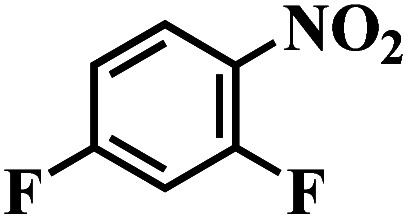	60 (9)	6.5	>99	>99
17	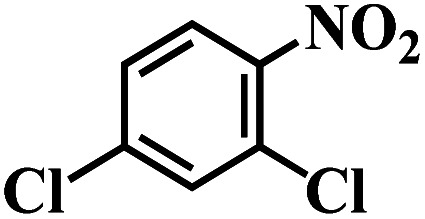	75 (31)	13	>99	>99
18	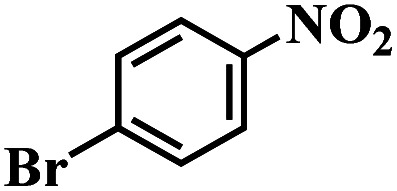	150 (127)	4.5	>99	>99
19	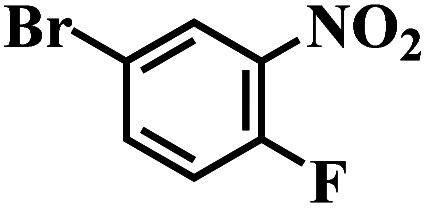	70 (19)	15.5	>99	>99
20	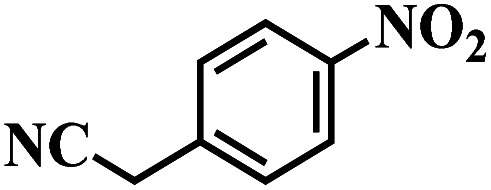	120 (116)	4.5	>99	>99
21	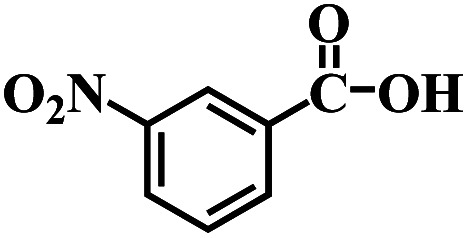	145 (142)	6.5	>99	>99
22	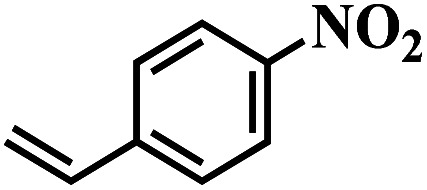	60 (20)	10	>99	>99
23	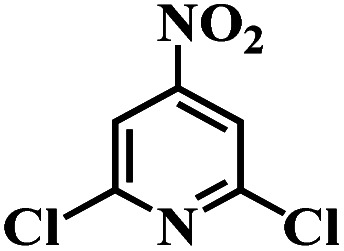	85 (57)	9	>99	91.5
24	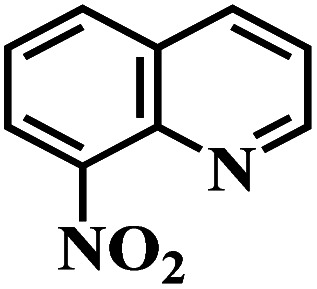	95 (92)	18	>99	>99
25	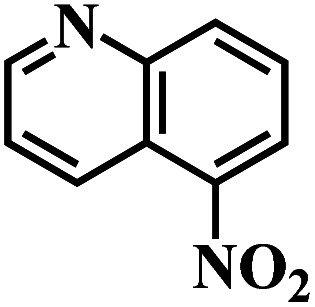	90 (73)	13.5	>99	>99

a Reaction conditions: 20 mg catalyst, substrates/Ru = 1000/1 (mol mol^−1^), H_2_ pressure = 4.0 MPa.

b Actual reaction temperatures and melting point of substrates in parentheses.

**Fig. 9:**
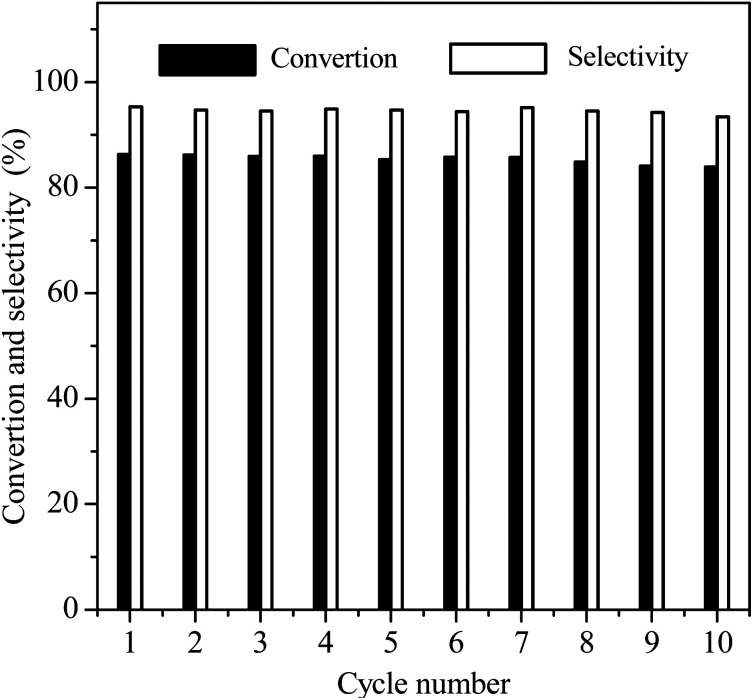
Reusable profiles of the 3Ru/OMC-800 for the hydrogenation of NB in the absence of solvent. Reaction conditions: 20 mg catalyst, NB/Ru = 1000/1 (mol mol^−1^), H_2_ pressure = 4.0 MPa, 50 °C; reaction time = 2 h.

**Fig. 10:**
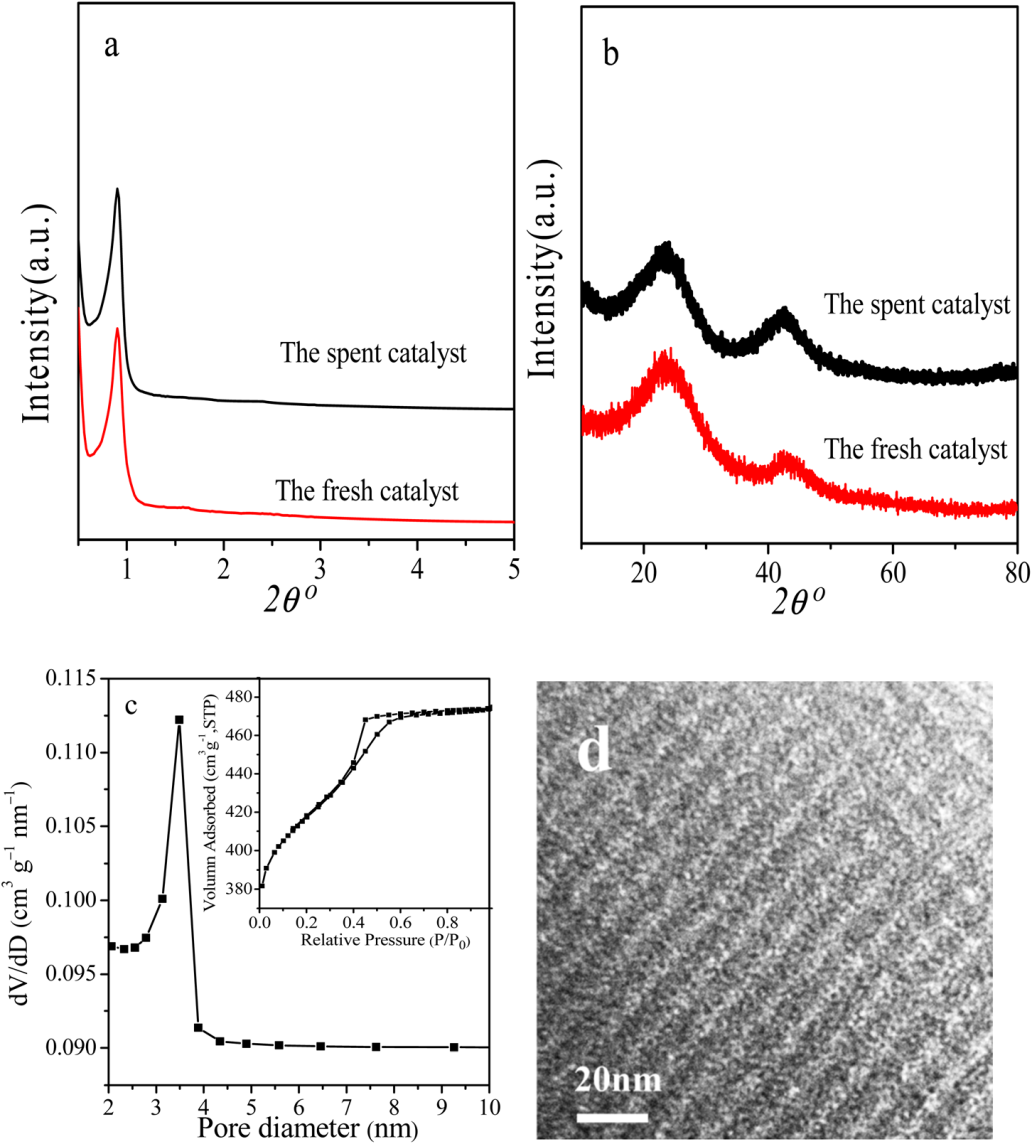
(a) Low-angle XRD, (b) wide-angle XRD patterns, (c) N_2_ sorption isotherms and pore size distribution and (d) TEM image of the spent catalyst for ten cycles.

Reusability is an important Index to evaluate the performance of heterogeneous catalysts. At last, we selected the reaction conditions with a conversion of ∼86% in the first cycle, at which the NB hydrogenation was controlled by chemical kinetics, to investigate the potential reusability of the 3Ru/OMC-800 catalyst for the hydrogenation of NB in the absence of solvent. The catalyst after each run was recovered by a simple filtration and drying without any other post-treatment. Ten reaction runs were performed and the NB conversion in each run was almost constant at ∼86%, as shown in Fig. 9. After the tenth run, the spent catalyst had no discernible variations in textural properties (*S*_BET_: 598.4 m^2^ g^−1^; *V*_p_: 0.39 cm^3^ g^−1^; *D*_p_: 3.5 nm), phase structure and Ru particle size distribution analyzed by XRD, BET and TEM as shown in Fig. 10. The final reaction solution and the spent catalyst was tested by ICP and it is found that there was no any Ru detected in the product and the metal content of the spent catalyst was almost the same with the fresh catalyst. This result demonstrated that the 3Ru/OMC-800 catalyst was highly stable and reusable for the solvent-free hydrogenation of nitroarenes with H_2_.

## 4. Conclusions

In summary, we have successfully prepared ultrafine Ru nanoparticles highly dispersed on ordered mesoporous carbon catalyst by a one-pot 8-HQ assisted multicomponent co-assembly method. The obtained Ru/OMC catalyst showed high activity and selectivity for the solvent free hydrogenation of various nitroarenes to the corresponding aromatic amines with excellent reusability. These results will also contribute to the development of efficient and environmentally friendly metal nanocatalysts for various organic reactions.

## Conflicts of interest

There are no conflicts to declare.

## Supplementary Material
